# A multiplanar complex resection of a low-grade chondrosarcoma of the distal femur guided by K-wires previously inserted under CT-guide: a case report

**DOI:** 10.1186/1471-2482-14-52

**Published:** 2014-08-13

**Authors:** Carmine Zoccali, Barbara Rossi, Virginia Ferraresi, Vincenzo Anelli, Alessandro Rita

**Affiliations:** 1 Oncological Orthopaedics Department, Muscular-skeletal Tissue Bank, IFO - Regina Elena National Cancer Institute, Via Elio Chianesi 53, Rome, Italy; 2 Medical Oncology “A”, IFO - Regina Elena National Cancer Institute, Via Elio Chianesi 53, Rome, Italy; 3 Radiological Department, IFO - Regina Elena National Cancer Institute, Via Elio Chianesi 53, Rome, Italy; 4 Orthopaedics and Traumatology Department, Policlinico “Umberto I”, University “Sapienza”, Rome, Italy

**Keywords:** Tumour resection, Osteotomy, Low-grade chondrosarcoma, Low-grade bone tumour, Wide margin, Navigation computer assisted

## Abstract

**Background:**

In muscular skeletal oncology aiming to achieve wide surgical margin is one of the main factors influencing patient prognosis. In cases where lesions are either meta or epiphyseal, surgery most often compromises joint integrity and stability because muscles, tendons and ligaments are involved in wide resection. When lesions are well circumscribed they can be completely resected by performing multi-planar osteotomies guided by computer-assisted navigation. We describe a case of low-grade chondrosarcoma of the distal femur where a simple but effective technique was useful to perform complex multiplanar osteotomies. No similar techniques are reported in the literature.

**Case presentation:**

A 57 year-old Caucasian female was referred to our department for the presence of a distal femur chondrosarcoma. A resection with the presenting technique was scheduled. The first step consists of inserting several K-wires under CT-scan control to delimitate the tumor; the second step consists of tumor removal: in operative theatre, following surgical access, k-wires are used as guide positioning; scalpels are externally placed to k-wires to perform a safe osteotomy.

**Conclusions:**

Computed assisted resections can be considered the most advantageous method to reach the best surgical outcome; unfortunately navigation systems are only available in specialized centres. The present technique allows for a multiplanar complex resection when navigation systems are not available. This technique can be applied in low-grade tumours where a minimal wide margin can be considered sufficient.

## Background

In muscular skeletal oncology aiming to achieve wide surgical margin is one of the main factors influencing patient prognosis [1]. In cases where lesions are either meta or epiphyseal, surgery most often compromises joint integrity and stability because muscles, tendons and ligaments are involved in wide resection. Moreover, prosthesis reconstruction is necessary in most cases but not sufficient to maintain preoperative activity levels [2].

When lesions are well circumscribed they can be completely and safely resected by performing multi-planar osteotomies guided by computer-assisted navigation. This technique was recently applied in select patients with sarcomas; however, the costs limit the procedure to being carried out in highly specialized centres where computer assisted navigation systems are available [3].

We report a case of low-grade chondrosarcoma of the distal femur where a new, safe, surgical technique permitted a minimally extended wide resection.

This technique could also be performed in a non-specialized centre where computer assisted navigation is not available, sometimes allowing to spare joint integrity and to reduce the need of prosthesis reconstruction, assuring improved functional outcome.

## Case presentation

**Figure 1:**
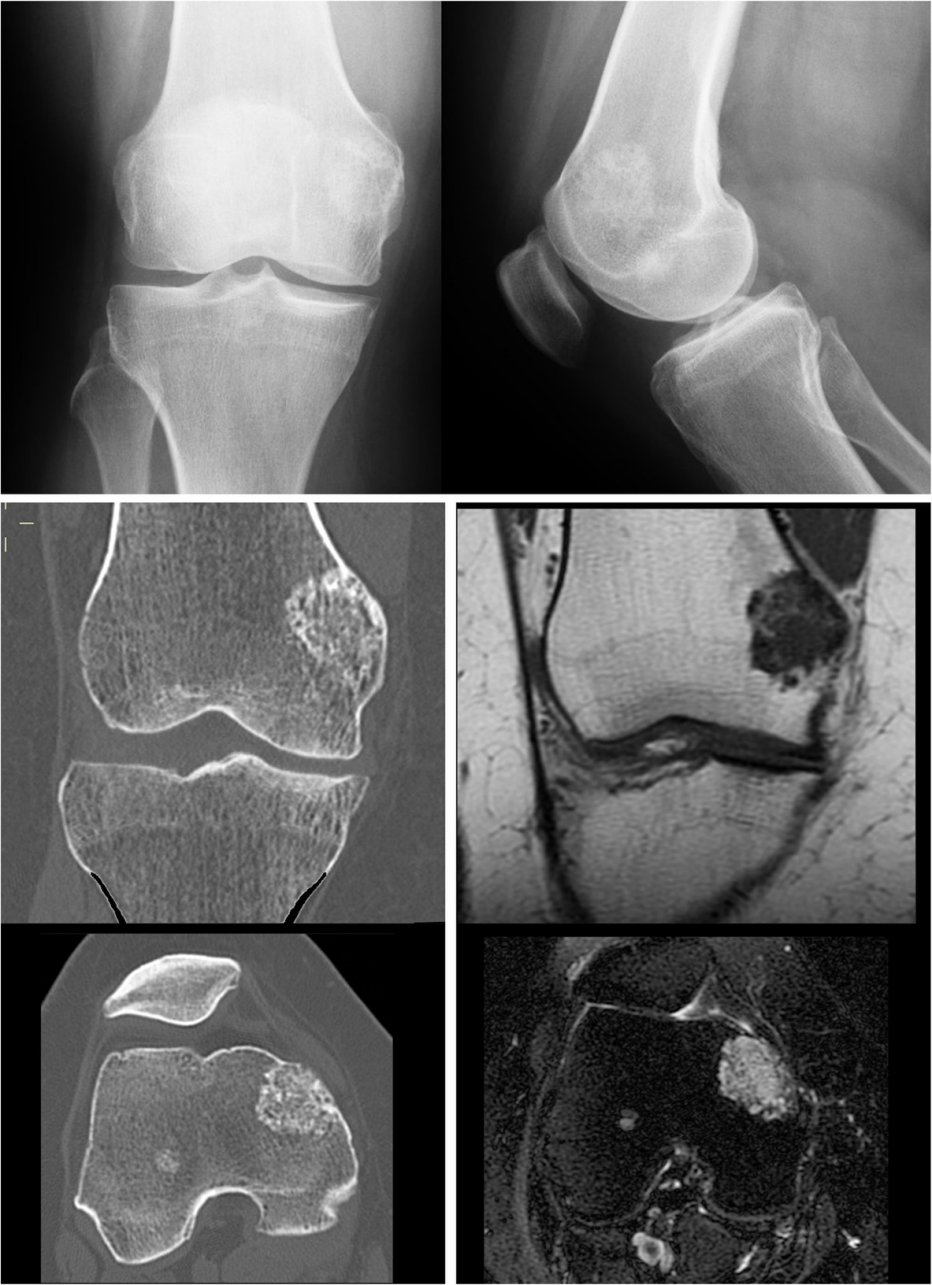
Preoperative imaging showing a sclerotic lesion located in the medial aspect of the medial condyle.

**Figure 2:**
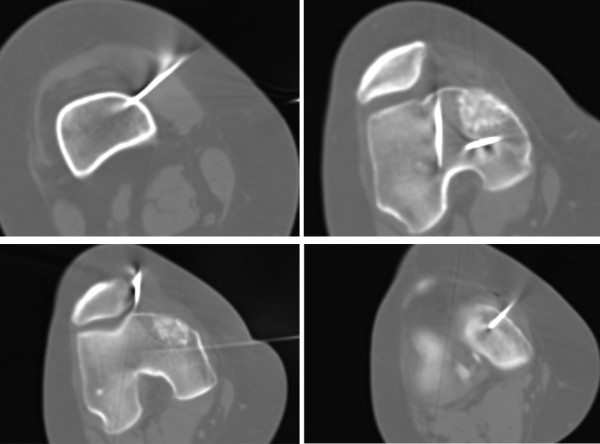
**CT scan showing the position of K-wires to define surgical margins.** In the upper left square the K-wire is inserted proximally to the lesion; in the upper right square two K-wires delimit the lesion laterally in the proximal part; in the lower left square two K-wires delimit the lesion laterally in the distal part; in the lower right the k-wire is inserted distally to the lesion.

**Figure 3:**
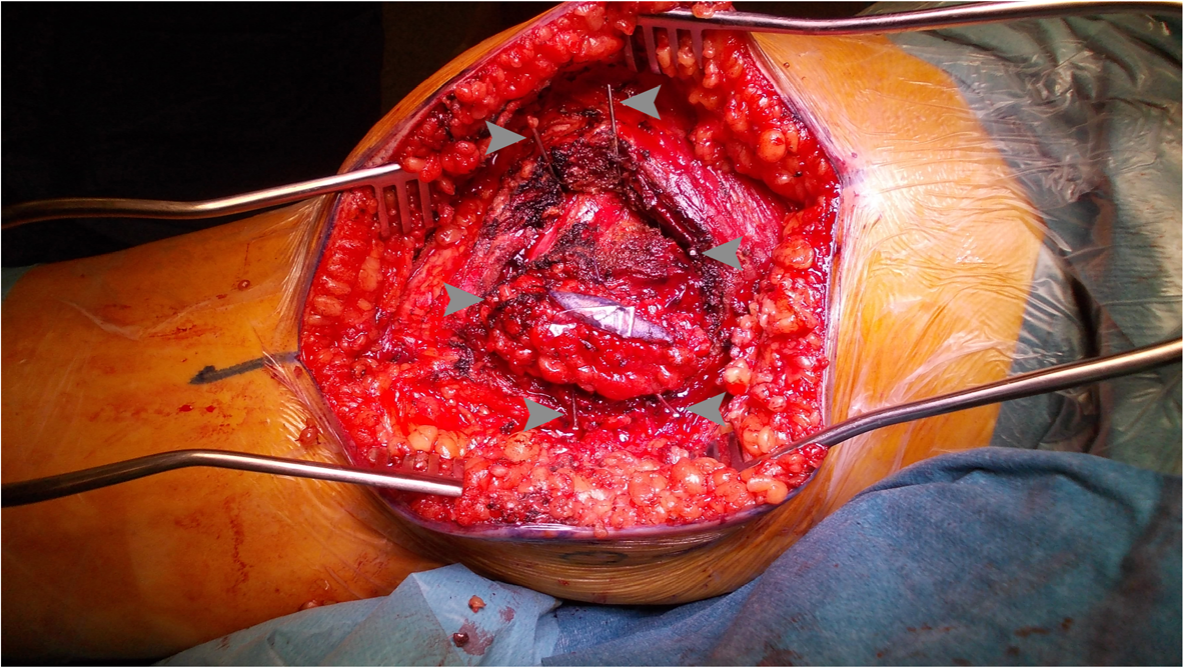
An intraoperative image showing the isolated mass delimited by the K-wires marked with the arrows.

**Figure 4:**
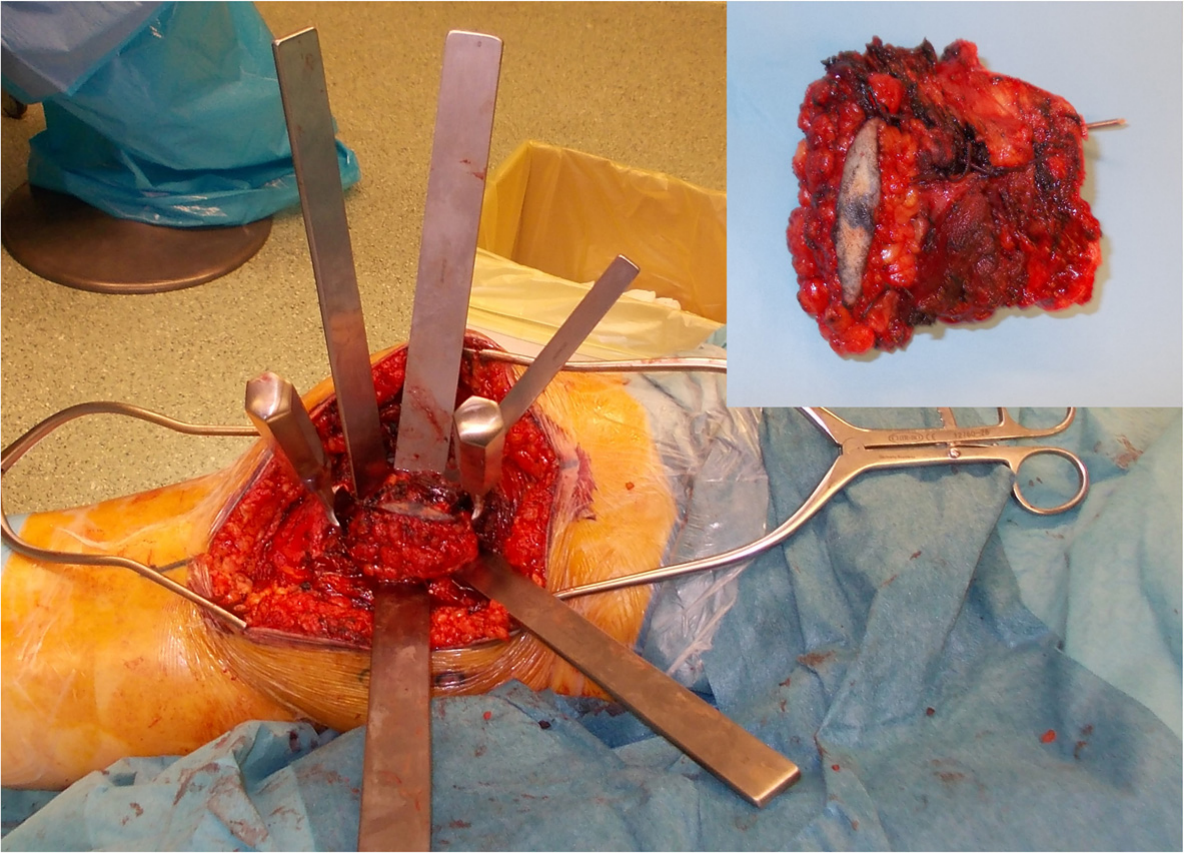
The scalpels were placed externally to the K-wires to perform multiplanar osteotomy; in the square the surgical specimen after resection with a k-wire resected en-bloc.

**Figure 5:**
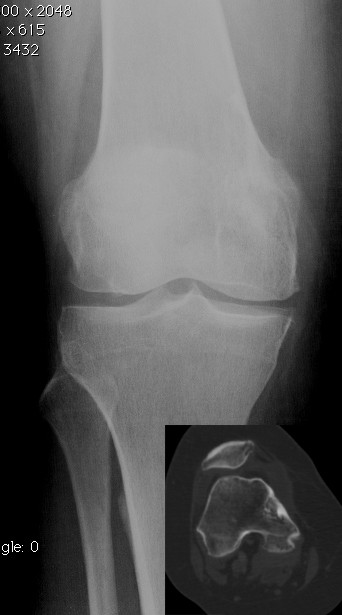
At two years follow-up, X-rays showing rather normal distal femurs; in the left square an immediate post-operative CT scan evidencing the wedge shaped osteotomy and rearrangement after a two year period.

A 57 year-old Caucasian female was referred to our department complaining of a pain in her right knee for the last six months. Imaging showed a non-homogeneous sclerotic lesion located in the medial aspect of the distal femur without cortical erosion resembling a benign chondroma (Figure 1). Due to local pain, aggravated by local tapping, a CT guided core biopsy was performed by medial access. The histology suggested a diagnosis of low-grade chondrosarcoma.Total-body CT-scan and PET-scan evidenced the absence of distant metastases. A distal femur resection and prosthesis reconstruction was proposed to the patient as curettage but, considering advantages and disadvantage and given that the tumour did not compromise the joint surface, we decided to program a complex osteotomy to preserve joint stability. Computerized tomography (CT) was used to plan the cutting planes to facilitate minimal wide resection (Figure 2).The day before surgery, under general anaesthesia, six K-wires were inserted, respecting 3-4 mm of margin, to delimit the tumour defining the resections planes, sparing joint surface and intra-articular space (Figure 2). The K-wires were then cut under the skin.The next day, in the operating theatre, the patient was placed in the supine position; to access the surgical area, the medial approach was used; the residual K-wires were identified and isolated externally until the bone surface (Figure 3). The scalpels were put externally to K-wires and used as a guide to perform four osteotomies, delimiting a wedge shape resection; the biopsy track was removed en-bloc with the mass (Figure 4). Reconstruction was performed by morcellized bone allograft. Histology, performed on the resection specimen, confirmed low grade chondrosarcoma and demonstrated wide resection with a minimum margin of four millimetres evidenced at the posterior osteotomy.At two year follow-up the X-ray shows a quite complete osteointegration (Figure 5), the patient is free from disease and able to walk normally with a modest patellar lateral instability. The Musculoskeletal Tumor Society score is 25; the patient is particularly satisfied to have maintained joint integrity.

The main objective in oncological surgical orthopaedics is to achieve a wide surgical margin; it is one of the most important prognostic factors [4]. Wide surgery can cause important loss of function due to ligaments, muscles, tendons and neurovascular bundles that can be involved in resection.

In low grade tumours obtaining minimal wide resection can allow the patient to maintain a satisfying quality of life. Computed assisted resections can be considered the most advantageous technique to reach the best surgical outcome; unfortunately, navigation systems are only available in specialized centres.

The reported case shows how it is possible to perform a multiplanar complex resection also when navigation systems are not available. This technique allowed resection of the mass, sparing the joint and collateral ligaments, sacrificing just medial patellar retinaculum and can be applied in low-grade tumours where a minimal wide margin can be considered sufficient.

The surgical treatment of low-grade chondrosarcoma of the appendicular skeleton remains controversial. Some authors prefer wide resection margins, while others consider intralesional curettage sufficient for adequate local control [5,6]. This discordance is probably caused by inter-observer variability in the histological diagnosis of cartilaginous tumours. Etchebehere et al. reported that biopsies yielded the correct diagnosis in 96% of chondrosarcoma cases; In actually, the correct grade was identified in only 46% of the time. This suggests that some chondrosarcomas may be undertreated using intralesional methods based on biopsy grading [7].

Our group sustains the use of wide resection for low-grade chondrosarcoma to minimize risk of local recurrence. We also maintain that to reduce the risk of undertreating a lesion that could be more aggressive than expected at definitive histology.

## Conclusions

The present technique allows for a safe multiplanar complex resection when navigation systems are not available. It can be applied in low-grade tumours where a minimal wide margin can be considered sufficient. More studies are advocated to verify its reliability.

## Consent

Written informed consent was obtained from the patient for publication of this Case report and any accompanying images. A copy of the written consent is available for review by the Editor of this journal.

## Competing interests

The authors declare that they have no competing interests.

## Authors’ contributions

ZC devised the technique, performed the surgery and has given final approval of the version to be published; RB participated to surgery and cared the patient follow-up; FV supervised the draft and cared the patient follow-up; AV performed the CT-guided K-wires insertion; RA wrote the manuscript. All authors read and approved the final manuscript.

## Pre-publication history

The pre-publication history for this paper can be accessed here:

http://www.biomedcentral.com/1471-2482/14/52/prepub
